# Goldenhar Syndrome Associated with Extensive Arterial Malformations

**DOI:** 10.1155/2015/954628

**Published:** 2015-11-25

**Authors:** Renee Frances Modica, L. Daphna Yasova Barbeau, Jennifer Co-Vu, Richard D. Beegle, Charles A. Williams

**Affiliations:** University of Florida, Gainesville, FL, USA

## Abstract

Goldenhar Syndrome is characterized by craniofacial, ocular and vertebral defects secondary to abnormal development of the 1st and 2nd branchial arches and vertebrae. Other findings include cardiac and vascular abnormalities. Though these associations are known, the specific anomalies are not well defined. We present a 7-month-old infant with intermittent respiratory distress that did not improve with respiratory interventions. Echocardiogram suggested a double aortic arch. Cardiac CT angiogram confirmed a right arch and aberrant, stenotic left subclavian artery, dilation of the main pulmonary artery, and agenesis of the left thyroid lobe. Repeat echocardiograms were concerning for severely dilated coronary arteries. Given dilation, a rheumatologic workup ensued, only identifying few weakly positive autoantibodies. Further imaging demonstrated narrowing of the aorta below the renal arteries and extending into the common iliac arteries and proximal femoral arteries. Given a physical exam devoid of rheumatologic findings, only weakly positive autoantibodies, normal inflammatory markers, and presence of the coronary artery dilation, the peripheral artery narrowings were not thought to be vasculitic. This case illustrates the need to identify definitive anomalies related to Goldenhar Syndrome. Although this infant's presentation is rare, recognition of specific vascular findings will help differentiate Goldenhar Syndrome from other disease processes.

## 1. Introduction

Goldenhar Syndrome (GS) or oculo-auricular-vertebral dysplasia (OAVD) is a rare condition characterized by typical ocular and auricular malformations. Associated findings may include cardiac and vascular anomalies. We present a 7-month-old infant with GS who had extensive vascular findings including internal carotid artery (ICA) agenesis, right aortic arch, and vascular ring, as well as coronary artery dilation and narrowing of the infrarenal aorta, common iliacs, and proximal femoral arteries. This is the first case report of an infant with GS with the novel findings of coronary artery dilation and narrowing of peripheral arteries, which may be confused with infantile vasculitis.

## 2. Case Report

This is a 7-month-old African American male born from a nonconsanguineous pregnancy to a 17-year-old primigravida who was group B strep and chlamydia positive and had spontaneous rupture of membranes. Pregnancy was remarkable for polyhydramnios and preterm labor resulting in premature SVD at 35 weeks' gestation. There was no medication or known teratogenic exposures or illnesses during pregnancy. At delivery, heart rate was initially 60 but improved with positive pressure ventilation. APGARs were 3, 8, and 9 at 1, 5, and 10 minutes, respectively.

The patient's family history was negative for any known birth defects. Both parents had normal craniofacial development. This is the only child from this couple but there are five normal paternal 1/2 siblings. The infant was transferred to our institution for evaluation of respiratory distress and congenital malformations. The newborn examination revealed left hemifacial microsomia, absence of left pinna, and difficulty opening the left eye raising concern for palsy. The right ear was normal as well as the cervical region without masses or sinuses. There was asymmetric crying faces; however, eyes were normal without epibulbar dermoids or coloboma of the eyelids. There was no obvious maxillary asymmetry. He had noisy upper airway respirations, coughing, and an abnormal cry reflecting tracheomalacia. The extremities had normal range of motion, joint, and muscle development without finger, toe, or nail abnormalities. Chest, abdomen, genitalia, and sacral areas appeared normal and the skin had no hemangiomas or birthmarks.

Bone conduction ABR was normal for each ear as well as normal air conduction testing of the right ear; however air conduction testing could not be performed in the left ear, given atresia of the external ear canal. He was also noted to have a soft 1/6 systolic murmur. An echocardiogram suggested a double aortic arch with a dominant right arch and mild branch pulmonary stenosis. A gated cardiac CT angiogram confirmed a right arch and an aberrant left subclavian artery that was markedly stenotic, dilation of the distal main pulmonary artery, and agenesis of the left thyroid lobe. Neonatal MRI of the brain demonstrated subarachnoid blood products but the brain was anatomically normal. Ophthalmological exam demonstrated findings consistent with retinopathy of prematurity.

Initial work-up by genetics showed a normal chromosome single nucleotide polymorphism (SNP) microarray, normal abdominal ultrasound, and babygram without evidence of spinal malformation or other bony abnormalities. Follow-up with genetics was recommended to monitor for growth and development; however family failed to present for outpatient appointments.

The infant presented to the emergency department and inpatient setting numerous times within the first several months of life with complaints of noisy breathing, stridor, and respiratory distress. He often required admission with viral illness that he acquired within the first year of life. During one admission, at age 7 months, he underwent bronchoscopy and was noted to have laryngotracheomalacia with dynamic collapse of the mid trachea, extrinsic compression of the mid trachea, and mild bronchomalacia of the left mainstem bronchus.

**Figure 1:**
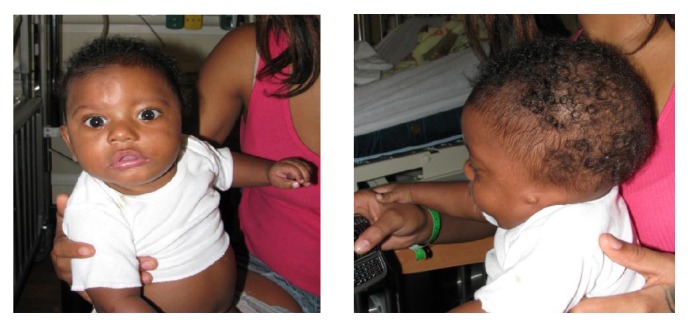
Patient with GS at 8 months of life with features of left hemifacial microsomia and left anotia.

**Figure 2:**
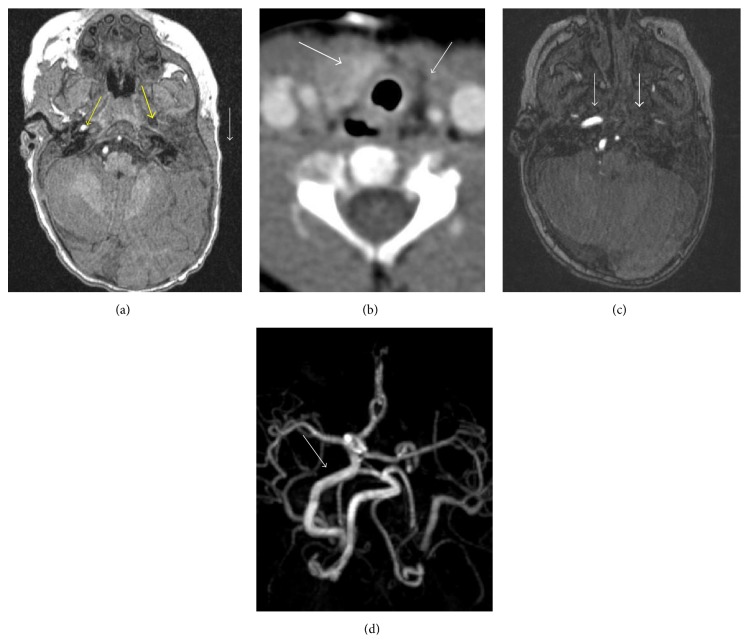
(a) Axial T1 weighted MR of the brain without contrast through the level of the external auditory canals demonstrates absence of the left external ear and external auditory canal (thin white arrow). There is also absence of the left internal carotid artery (thick yellow arrow). The right internal carotid artery is shown for comparison (thin yellow arrow). (b) Axial postcontrast CT of the neck at the level of the thyroid gland shows a normal right thyroid lobe (thick white arrow) with absence of the left thyroid lobe (thin white arrow). (c) Axial noncontrast time of flight MRA of the brain demonstrates a normal right internal carotid artery at its petrous segment (thin white arrow). There is absence of the left internal carotid artery (thick white arrow). (d) Three-dimensional reformation of the MRA of the brain demonstrates a normal right internal carotid artery (thin white arrow). The left internal carotid artery is absent.

He was reevaluated by genetics at 8 months of age. He had anotia on the left with only a nubbin of tissue present, without pits or accessory tags (Figure 1). A repeat MRI of the brain (Figure 2) demonstrated complete absence of the auricle and external auditory canal, and dysmorphic incus and malleus within the internal ear. Unilateral and ipsilateral absence of the left internal carotid artery with absence of the carotid canal typical of GS were also noted. MRI of the head and neck also confirmed left thyroid agenesis and absence versus hypoplasia of the left trigeminal nerve.

**Figure 3:**
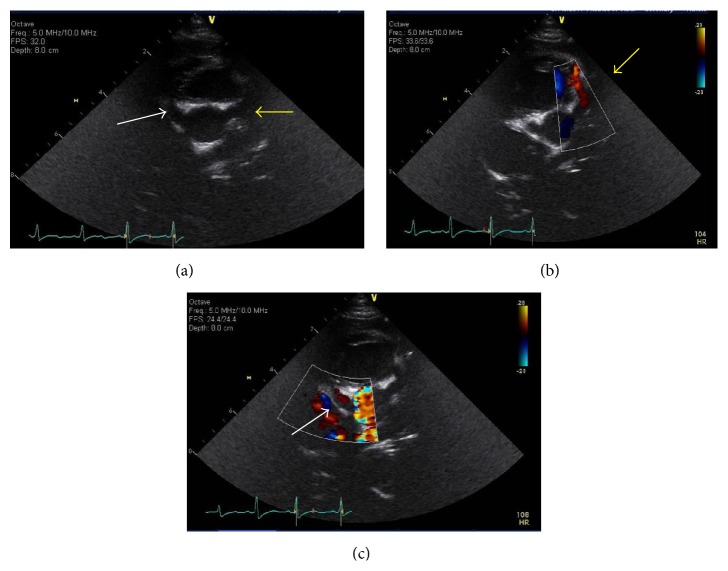
Presurgical transthoracic echocardiographic images of the right and left coronary arteries. (a) 2D transthoracic echocardiographic coronary images prior to cardiac surgery demonstrate dilated proximal right coronary artery (white arrow) and left main coronary artery (yellow arrow). (b) 2D and color Doppler transthoracic image of the dilated left main and left anterior descending coronary arteries (yellow arrow). (c) 2D and color Doppler transthoracic image of the dilated right coronary artery (white arrow).

**Figure 4:**
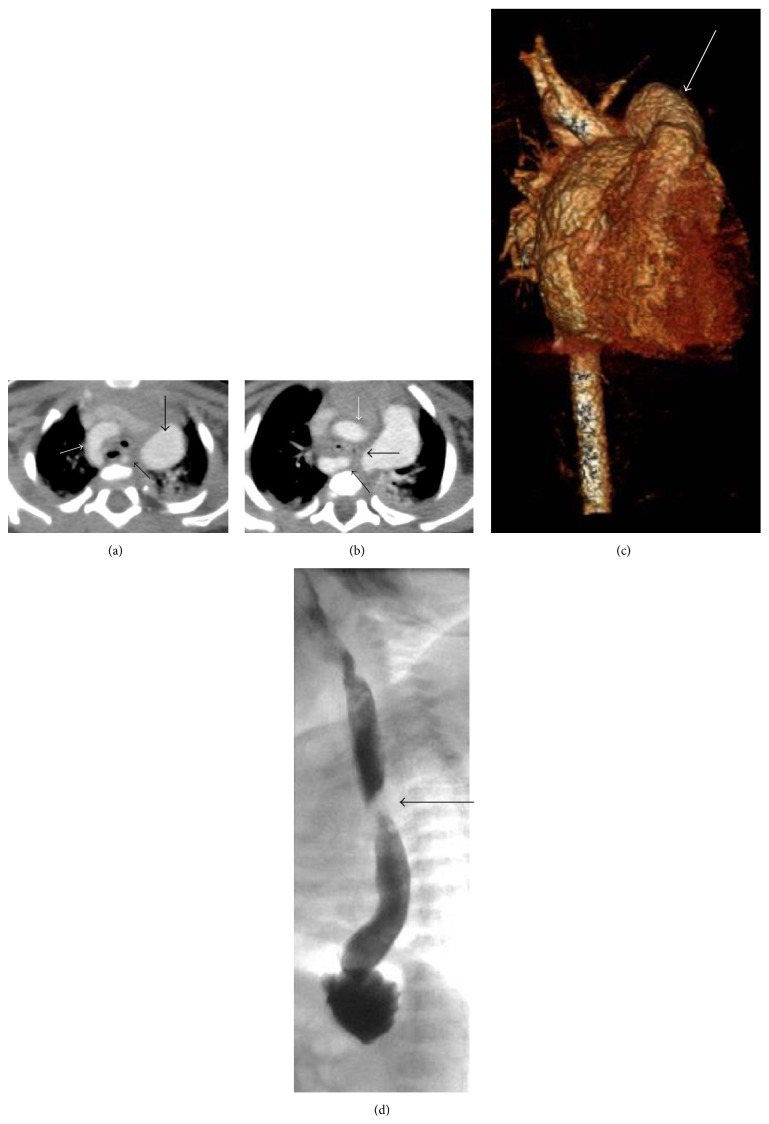
(a) Axial postcontrast CTA of the chest demonstrates a right aortic arch (white arrow). The aberrant left subclavian artery is seen extending posterior to the esophagus (thin black arrow) which helps form the complete vascular ring. The pulmonary artery was enlarged in this patient (thick black arrow). (b) Axial postcontrast CTA of the chest at a slice inferior to (a) demonstrates the ascending aorta projecting to the right (white arrow) and the aberrant left subclavian artery arising from the descending aorta and traveling posterior to the esophagus (thin black arrow). The complete vascular ring causes mass effect and narrowing of the esophagus and trachea (thick black arrow). (c) 3D surface rendered reformation of the CTA of the chest viewed in the anteroposterior dimension clearly demonstrates the right aortic arch (thick white arrow). (d) Fluoroscopic image of a barium esophagram demonstrates mass effect on the posterior esophagus (thick black arrow) created by the aberrant left subclavian artery.

**Figure 5:**
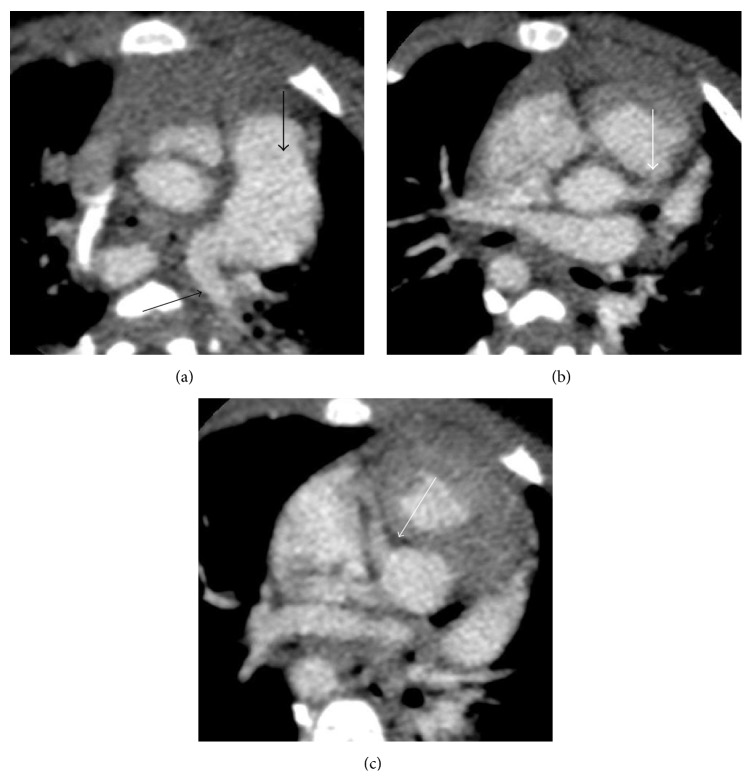
Multiple slices from a postcontrast CTA of the chest from superior to inferior demonstrate enlargement of the pulmonary artery (thick black arrow) and crisscross pattern of the pulmonary arteries (thin black arrow). There is dilatation of the origins of the left coronary artery (thick white arrow) and right coronary artery (thin white arrow).

From a cardiovascular standpoint, at 8 months of age, two follow-up echocardiograms showed severely dilated right and left coronary artery systems in addition to previously identified cardiac anomalies. No pericardial effusion was seen on either echo. The proximal right coronary artery measured 0.3–0.4 cm (*Z*-scores 4.98–9.3). The left main coronary artery diameters were 0.38 cm–0.43 cm (*Z*-scores of 6.14–6.97) (Figure 3). Follow-up CT angiogram of the chest (Figures 4 and 5) confirmed right aortic arch with stenotic and aberrant left subclavian artery forming a complete vascular ring compressing the lower esophagus. There were diminutive proximal right and left bronchi, “crisscrossed” branch pattern of the pulmonary arteries (the left pulmonary artery arises from the main pulmonary artery and more superiorly than the right pulmonary artery). The right and left coronary arteries are diffusely dilated with normal origins and without fistula.

**Figure 6:**
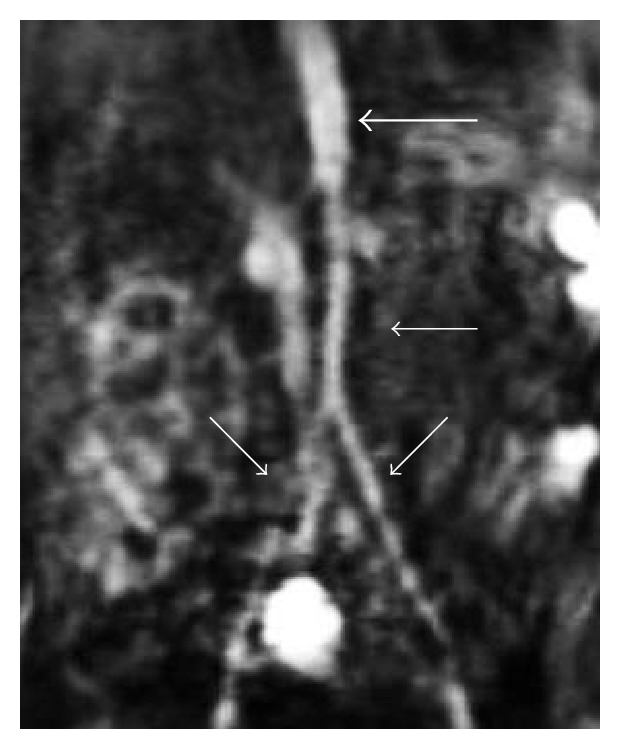
Coronal view of a postcontrast MRA of the abdomen demonstrates normal caliber of the abdominal aorta above the renal arteries (thick white arrow). There is symmetric narrowing of the abdominal aorta below the level of the renal arteries extending into the iliac arteries (thin white arrows). Of note, the beaded appearance of the iliac arteries is artifactual.

Given his dilated coronary arteries, pediatric rheumatology was consulted to evaluate for vasculitic disease. No historical or physical findings for Kawasaki's disease, Lupus, or other vasculitic disorders were noted including lack of fever, lymphadenopathy, hepatosplenomegaly, edema, rash, arthritis, asymmetric pulses, mucocutaneous findings, nailbed telangiectasia, digital ulcers, Raynaud's phenomenon, bruits, or ocular injection. However, given the lack of explanation for his coronary artery dilation, further work-up was recommended. Notably, he was found to have weakly positive ANA (1 : 40, speckled) and weakly positive Smith antibody (26 units) on autoimmune testing. His other autoantibody screening demonstrated negative P-ANCA, C-ANCA, dsDNA, SM-RNP antibody, SS-A and SS-B, and anti-cardiolipins IgG and IgM. His von willebrand antigen, DRVVT, PT, PTT, CRP, ESR, C3, C4, and quantitative immunoglobulins were all normal. MRA of the abdomen and pelvis (Figure 6) was performed and demonstrated symmetric narrowing without beading or inflammation of the aorta below the renal arteries and extending into the common iliac arteries and proximal femoral arteries. Upper and lower extremity deep venous ultrasounds were also found to be normal. Upon retrospective review of his neonatal echocardiogram he did have dilated coronary arteries at birth. Given the lack of physical exam features for rheumatic disease, only weakly positive autoantibodies, normal inflammatory markers, and congenital presence of the coronary artery dilation, the peripheral artery narrowing were not thought to be vasculitic in nature.

**Figure 7:**
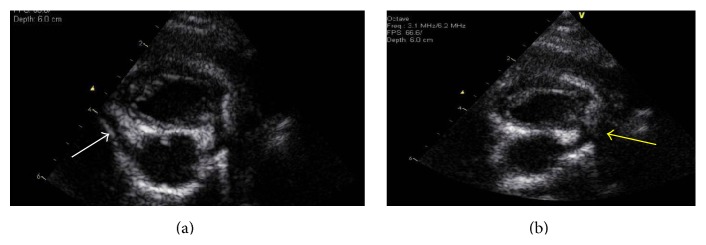
Postsurgical transthoracic echocardiographic images of the right and left coronary arteries. (a) 2D and color Doppler transthoracic image of the persistently dilated right coronary artery (white arrow). (b) 2D and color Doppler transthoracic image of the persistently dilated left main coronary artery (yellow arrow).

The infant had repair of the vascular ring at 9 months of age without complications however; he has continued to demonstrate stridor at baseline, likely from his laryngotracheomalacia. Postsurgical echocardiograms continued to show dilation of his coronary arteries. The repeat right coronary artery diameters by echo were 0.28–0.33 cm (*Z*-scores of 4.47–5.94) and the left middle coronary artery diameters were 0.35 cm–0.39 cm (*Z*-scores 4.52–5.47), which are similar to his previous studies (Figure 7).

## 3. Discussion

GS is generally thought to be due to a developmental abnormality of 1st and 2nd branchial arches and vertebral bodies resulting in the triad of craniofacial microsomia and ocular and vertebral abnormalities. Given this typical triad, it has also become known as oculo-auriculo-vertebral dysplasia (OAVD) [1]. The diagnosis of this condition is mostly based on these characteristic clinical findings but supportive radiologic and laboratory results can be helpful. Ophthalmologic and otorhinolaryngologic examinations are also important [12]. GS occurs in conjunction with cardiac and vascular anomalies although no single cardiac or vascular anomaly is characteristic. Tetralogy of Fallot (TOF) and ventricular septal defects (VSD) are the most common cardiac anomalies reported and agenesis of the internal carotid artery (ICA) is one of the more common vascular anomalies [2]. ICA agenesis ipsilateral to the hemifacial microsomia is more common than contralateral [6].

Despite being classically described as a craniofacial disorder involving bilateral or hemifacial underdevelopment and ocular changes, other less proximate abnormalities have been reported since its initial description. These include other organ systems such as cardiopulmonary [2–5, 7], vascular [2–6], and renal and genitourinary abnormalities [5, 9–11] (Table 1).

A literature review of GS syndrome does report unilateral ICA findings as well as vascular ring, but, to our knowledge, there are no reports of GS with such extensive arterial abnormalities as described in this infant [5, 6]. Due to the presence of the associated other cardiac abnormalities typical of GS as well as the extensive and symmetric nature of these arteriographic findings, we think that the vascular changes in our patient are probably part of the GS disorder. In our case there was no correlation of the laterality of the vascular findings to the facial deformities, which has been reported in other less typical findings of GS. It is likely that patients with GS may have peripheral vascular changes but MRAs have not been routinely ordered on these patients due to lack of clinical need or suspicion. Of note, Rad had reported one case of bilateral renal artery stenosis that was limited without extension distally [5].

In spite of our patient's extensive findings of abnormal vasculature, his prenatal exam only demonstrated slight murmur. Otherwise, his pulses were normal and vascular exam was without bruits. The MRA was ordered looking for vasculitis due to the presence of dilated coronary arteries without clear explanation such as a fistula. Patients' vasculitides that present in infancy with coronary artery involvement include Polyarteritis Nodosa (PAN), systemic onset juvenile arthritis (SOJIA), and Kawasaki disease (KD) [8, 13–15]. PAN is a non-ANCA associated necrotizing inflammation of the medium sized blood vessels that typically presents with skin ulcerations, arthritis, GI vasculitis, nephritis, and orchitis as well as anemia, thrombocytosis, and elevated inflammatory markers. On angiography, the vasculitis is frequently segmental and may form microaneurysms. Patients with SOJIA may have dilated coronary arteries but typically have quotidian fevers, salmon colored evanescent migratory rash, lymphadenopathy, hepatosplenomegaly, and inflammatory CBC as well as arthritis. In Kawasaki disease patients typically present with fever, mucocutaneous changes, lymphadenopathy, ocular injection, and dilated coronary arteries with vascular brightness.

GS has associated malformations that have yet to be explained by one theory [16]. Chromosomal abnormalities, vascular pathogenesis that disturbs placental or embryonic blood supply, disturbance of neural crest cells, environmental influences, teratogens, and maternal diabetes have all been suggested as possible etiologies; however, the cause of GS remains unknown [12, 16–20]. Family reports on occasion have suggested autosomal dominant or recessive inheritance [10, 16, 19]. Chromosome disorders or single gene defects have been implicated but not proven as causative of the syndrome. Affected children typically have normal chromosomes and normal family histories and recurrence in a family is rare. This suggests that GS may be a stochastic, spontaneous event rather than an inherited one. Sometimes teratogens such as thalidomide, primidone, tamoxifen, cocaine, and retinoic acid have been implicated and there does seem to be an increased incidence of GS among the children of Gulf War veterans [19].

With regard to theories of vascular pathogenesis, Ottaviano et al. state that there is a close link between the structures from where the carotid vessels and the ear structures originate [20]. They state that the vascular malformations might derive from a vascular deficiency of the cephalic mesodermal cells with subsequent alteration of the development of the I and II branchial arches. Experiments in lambs from Escobar and Liechty demonstrated a possible link between late gestational vascular disruptions and subsequent craniofacial anomalies [21]. They showed that interruption of the carotid blood flow in the late gestation period produces phenotypic craniofacial anomalies similar to those seen in GS [20, 21]. They suggest that the early lack of blood flow in the cephalic region at the I and II branchial arches could influence the appearance of the OAV spectrum.

It is unclear what led to the more pervasive vascular abnormalities in our patient involving narrowing of the infrarenal aorta, common iliac arteries, and proximal femoral arteries. Although the etiology is unclear, peripheral arterial narrowing and coronary artery dilation may be a new reportable finding associated with GS. These findings may warrant the need for more extensive vascular screening for patients with GS.

## Figures and Tables

**Table 1:** 

System [ref.]	Description of finding	Findings unique to this case
Auricular [1]	Anotia, microtia, conductive hearing loss, and preauricular skin tags	

Ophthalmologic [1]	Epibulbar dermoids and coloboma of the eyelids	

Facial [1]	Hemifacial microsomia and cleft lip and palate	Severe microtia in the absence of overt hemifacial microsomia

Cardiac [2–5]	(i) Tetralogy of Fallot (ii) Ventricular septal defect (iii) Abnormalities of the aortic arch (hypoplasia, right aortic arch, right circumflex aortic arch, vascular ring, coarctation of the aorta, and aberrant right subclavian artery) (iv) Complete transposition of great arteries (v) Persistent Patent Ductus Arteriosus (isolated or in combination with other anomalies) (vi) Isolation of the left innominate artery with bilateral Patent Ductus Arteriosus (vii) Dextrocardia (viii) Dysplastic valves	Coronary artery dilation

Vascular malformations [2–6]	(i) Agenesis of the internal carotid artery (ii) Hypoplastic external carotid arteries (iii) Persistent left superior vena cava (iv) Isolated left innominate artery (v) Vascular ring (vi) Bilateral renal artery stenosis (vii) Hypoplastic pulmonary artery and its branches (viii) Aberrant right subclavian artery	(i) Narrowing of infrarenal aorta, common iliacs, and proximal femoral arteries (ii) Crisscross branch pattern of the pulmonary arteries

Pulmonary [2, 7, 8]	(i) Incomplete lobulation (ii) Unlobed lungs (iii) Hypoplasia of the lung (typically on the ipsilateral side of the facial anomalies) (iv) Pulmonary hypoplasia and agenesis (v) Laryngotracheomalacia	

Renal [5, 9]	(i) Ectopic and/or fused kidneys (ii) Multicystic kidney	

Genitourinary [9–11]	(i) Ureteropelvic junction obstruction (ii) Ureteral duplication (iii) Vesicoureteral reflux	

## References

[1] Ashokan C. S., Sreenivasan A., Saraswathy G. K. (2014). Goldenhar syndrome—review with case series. *Journal of Clinical and Diagnostic Research*.

[2] Pierpont M. E. M., Moller J. H., Gorlin R. J., Edwards J. E. (1982). Congenital cardiac, pulmonary, and vascular malformations in oculoauriculovertebral dysplasia. *Pediatric Cardiology*.

[3] Morrison J., Mulholland H. C., Craig B. G., Nevin N. C. (1992). Cardiovascular abnormalities in the oculo-auriculo-vertebral spectrum (Goldenhar syndrome). *American Journal of Medical Genetics*.

[4] Digilio C. M., Calzolari F., Capolino R. (2008). Congenital heart defects in patients with oculo-auriculo-vertebral spectrum (Goldenhar syndrome). *American Journal of Medical Genetics Part A*.

[5] Rad E. M. (2014). Goldenhar syndrome with right circumflex aortic arch, severe coarctation and vascular ring in a twin pregnancy. *Annals of Pediatric Cardiology*.

[6] Ventura E., Ormitti F., Crisi G., Sesenna E. (2014). Goldenhar syndrome associated with contralateral agenesis of the internal carotid artery. *The Neuroradiology Journal*.

[7] Jacobs W., Vonk Noordegraaf A., Golding R. P., van den Aardweg J. G., Postmus P. E. (2007). Respiratory complications and Goldenhar syndrome. *Breathe*.

[8] Ozen S., Pistorio A., Iusan S. M. (2010). EULAR/PRINTO/PRES criteria for Henoch-Schönlein purpura, childhood polyarteritis nodosa, childhood Wegener granulomatosis and childhood Takayasu arteritis: Ankara 2008. Part II: final classification criteria. *Annals of the Rheumatic Diseases*.

[9] Soni N. D., Rathod D. B., Nicholson A. D. (2012). Goldenhar syndrome with unusual features. *Bombay Hospital Journal*.

[10] Mutanabbi M., Rahman M. A., Mamun A. A., Helal M. A., Billah M. B., Islam K. A. (2014). Goldenhar syndrome—a case report. *Mymensingh Medical Journal*.

[11] Ritchey M. L., Norbeck J., Huang C., Keating M. A., Bloom D. A. (1994). Urologic manifestations of Goldenhar syndrome. *Urology*.

[12] Pinheiro A. L. B., Araújo L. C., Oliveira S. B., Sampaio M. C. C., Freitas A. C. (2003). Goldenhar's syndrome—case report. *Brazilian Dental Journal*.

[13] Stone J. H., Imboden J. B., Hellman D. B., Stone J. H. (2013). Polyarteritis nodosa. *CURRENT Diagnosis & Treatment Rheumatology*.

[14] Kawakami T. (2012). A review of pediatric vasculitis with a focus on juvenile polyarteritis nodosa. *American Journal of Clinical Dermatology*.

[15] Canares T. L., Wahezi D. M., Farooqi K. M., Pass R. H., Ilowite N. T. (2012). Giant coronary artery aneurysms in juvenile polyarteritis nodosa: a case report. *Pediatric Rheumatology*.

[16] Hartsfield J. K. (2007). Review of the etiologic heterogeneity of the oculo-auriculo-vertebral spectrum (Hemifacial Microsomia). *Orthodontics & Craniofacial Research*.

[17] Preis S., Majewski F., Hantschmann R., Schumacher H., Lenard H. G. (1996). Goldenhar, Möbius and hypoglossia-hypodactyly anomalies in a patient: syndrome or association?. *European Journal of Pediatrics*.

[18] Sadler T. W., Rasmussen S. A. (2010). Examining the evidence for vascular pathogenesis of selected birth defects. *American Journal of Medical Genetics Part A*.

[19] Sharma J. K., Pippal S. K., Raghuvanshi S. K., Shitij A. (2006). Goldenhar-Gorlin's syndrome: a case report. *Indian Journal of Otolaryngology and Head & Neck Surgery*.

[20] Ottaviano G., Calzolari F., Martini A. (2007). Goldenhar syndrome in association with agenesia of the internal carotid artery. *International Journal of Pediatric Otorhinolaryngology*.

[21] Escobar L. F., Liechty E. A. (1998). Late gestational vascular disruptions inducing craniofacial anomalies: a fetal lamb model. *Journal of Craniofacial Genetics and Developmental Biology*.

